# Examination of Pork for Trichinae at Hamburg*Wochenschrift für Thierheilkunde, No. 20, 1884.

**Published:** 1884-10

**Authors:** 


					﻿Examination of Pork for Trichinae at Hamburg.*
fl "fl	o fl -d
No of	o 2 ® =	® ® ®cc
Examiners.	Trichinous.	Trichinous.
8l^	6	2
H	H
1881—	60	73113	695.=0.95per et. 55799	2.004 per cent.
1882—	48	18619	l75.=0.95per ct.	60527	None.
1883—	51	18315	92.=0.69per ct.	62936	None.
*Wochenschrift fur Thierheilkunde, No. 20, 1884.
				

